# Patient safety in elderly hip fracture patients: design of a randomised controlled trial

**DOI:** 10.1186/1472-6963-11-59

**Published:** 2011-03-21

**Authors:** Hanneke Merten, Sanne Lubberding, Inge van Wagtendonk, Paul C Johannesma, Cordula Wagner

**Affiliations:** 1 NIVEL, Netherlands institute for health services research, PO Box 1568, 3500 BN Utrecht, The Netherlands; 2 Department of Trauma Surgery, VU university medical center, De Boelelaan 1117, 1081 HV Amsterdam, The Netherlands; 3 Department of Public and Occupational Health, EMGO Institute for Health and Care Research, VU university medical center, Van der Boechorststraat 7, 1081 BT Amsterdam, The Netherlands

## Abstract

**Background:**

The clinical environment in which health care providers have to work everyday is highly complex; this increases the risk for the occurrence of unintended events. The aim of this randomised controlled trial is to improve patient safety for a vulnerable group of patients that have to go through a complex care chain, namely elderly hip fracture patients.

**Methods/design:**

A randomised controlled trial that consists of three interventions; these will be implemented in three surgical wards in Dutch hospitals. One surgical ward in another hospital will be the control group. The first intervention is aimed at improving communication between care providers using the SBAR communication tool. The second intervention is directed at stimulating the role of the patient within the care process with a patient safety card. The third intervention consists of a leaflet for patients with information on the most common complications for the period after discharge. The primary outcome measures in this study are the incidence of complications and adverse events, mortality rate within six months after discharge and functional mobility six months after discharge. Secondary outcome measures are length of hospital stay, quality and completeness of information transfer and patient satisfaction with the instruments.

**Discussion:**

The results will give insight into the nature and scale of complications and adverse events that occur in elderly hip fracture patients. Also, the implementation of three interventions aimed at improving the communication and information transfer provides valuable possibilities for improving patient safety in this increasing patient group. This study combines the use of three interventions, which is an innovative aspect of the study.

**Trial registration:**

The Netherlands National Trial Register NTR1562

## Background

In many Western countries the population is ageing rapidly. This change in population demographics makes the necessary adjustments in healthcare needs and requirements for this group an increasingly important issue in research and policy [1]. Besides the expected shortages in healthcare staff, the specific clinical challenges that have to be dealt with in elderly patients are important. Elderly patients often do not show typical signs and symptoms of a disease, thus making a timely and accurate diagnosis more difficult [2]. In addition, these patients are often suffering from substantial co-morbidity, which further complicates the treatment.

In accordance with the changing demographics of the general population, the incidence of hip fractures will also increase in the upcoming years [3]. Hip fracture patients represent a clinical management challenge; they are an increasing group of predominantly older persons with a variety of medical co-morbidities under varying levels of control. In addition, they are facing operative repair of the fracture and frequently a difficult postoperative rehabilitation [4]. Even after this long rehabilitation, only half of these patients will return to their community and only about a third regains their former level of function [5,6]. Research has shown that hip-fractures are associated with an one-year mortality as high as 14 to 36% [6]. Also, the treatment of a hip fracture is very expensive [7].

The care process for hip fracture patients has been classified as highly complex. To illustrate the multidisciplinary treatment; a case study in a university hospital in Sweden showed that hip fracture patients initially meet doctors, nurses and other staff in the Emergency Department (ED), radiology, surgery, geriatrics, anaesthesiology, the operating room, and sometimes the Intensive Care Unit (ICU) [8]. The involvement of many different specialties makes optimal communication, hand-offs and information transfer even more important. To minimise the risk of complications, a complex series of interventions is required, several physician specialties and hospital departments need to be coordinated, and time is of the essence. Unfortunately, this level of coordination seems difficult to achieve [4]. Structuring the information transfer and communication can be an important step in this process.

The study we describe in this protocol focuses on the effects of improving the quality and safety of hip fracture patients by structuring the information transfer and interprofessional communication. An intervention study is set up to implement three interventions in a combined manner and is aimed at 1) improving the clinical communication between care providers with the introduction of the SBAR-communication tool, 2) strengthening the role of patients and their families with a patient safety pocket card and 3) providing patients and their families with specified information for the period after discharge by handing out a leaflet which describes the most common complications and who to contact with specific questions. One of the goals of this study is to establish whether this combined intervention programme can result in a reduction of the complication and adverse event (AE) rate in elderly hip fracture patients.

A complication is an unintended and unwanted event or state during or following medical specialist treatment, that has an unfavourable effect on the health of the patient to such an extend that adjustment of the medical treatment is necessary or that irreparable harm has occurred [9]. An Adverse Event (AE) is defined as an unintended injury that results in temporary or permanent disability, death or prolonged hospital stay, and is caused by healthcare management rather than by the patient's underlying disease process [10-13]. De Vries et al. (2008) recently conducted a systematic review based on eight patient record review studies to calculate the mean overall incidence of in-hospital AEs. They stated that in 9.2% of all hospital admissions one or more AEs occurred. Of these AEs nearly half (43.5%) were preventable and 7.4% contributed to death [14]. Other research showed that elderly patients are at increased risk of suffering from Adverse AEs [15,16]. To our knowledge, there is no published material on the AE rate in elderly hip fracture patients yet.

AEs are generally caused by a combination of factors. These often involve organisational, human, technical and patient-related factors [17,18]. One of the contributing human-related factors are communication failures [19], they are estimated to be of major influence in 60-70% of serious incidents [20-22]. There are many barriers that can potentially contribute to communication difficulties between clinicians. Some examples are: a lack of structure, differences in communication styles and uncertainty about who is responsible for the patient's care [23-25]. Several tools have been developed to structure the communication between care providers. One of these is called SBAR, a situational briefing tool developed by Kaiser Permanente [19,24]. SBAR stands for Situation, Background, Assessment, Recommendation and serves as a model that care providers can use to structure clinical communication. So far, to our knowledge, only a limited amount of published work exists on the effectiveness of the SBAR-tool on improving clinical communication [22,24]. The SBAR-tool could have the potential to improve communication between care providers about patients when effective in the clinical setting, so further evidence is needed. This can benefit the care for our complex patient group where a timely and accurate transfer of information is essential.

Another important factor for patient safety is improving the communication between care-providers and patients. Previous findings provide some support for the view that patients are willing to be more involved when it comes to reducing patient safety incidents [26]. However, some research has also shown that this need for patient involvement varies for several demographic variables, such as age. Younger patients tend to want more involvement than older patients. [26-29]. It can therefore be useful to stimulate elderly patients to get more actively involved in their own care process as they are the only one to be involved in the care process from beginning to end. Of course, an important question is to what extend elderly patients actually want to be involved and whether they feel comfortable enough to actively question the (safety) practices of health care staff. It is also important that patients should never feel responsible for the safety of the care they receive.

A third issue related to the previous two is the lack of information that patients can experience, especially during and after discharge. Smit et al. (2005) showed that patients, in their own opinion, do not receive enough information on what they have to do in an emergency situation, how to use several medications together and what they are allowed to do after discharge [30]. Often, patients are discharged from hospital and come back to the outpatient clinic after a scheduled amount of time. In between, it is not always clear to them who to turn to with questions following the hospital admission. An adequate and timely transfer of information about the hospital admission towards the general practitioner (GP) is sometimes lacking so the GP is not completely informed about the current medical condition of the patient [31,32]. This lack of information transfer can make it difficult to contact the appropriate care provider with specific questions, even though early detection and treatment of possible problems are very important.

### Aim and research questions

The aim of this randomised controlled trial (RCT) is to evaluate the effectiveness of a patient safety intervention programme for elderly hip fracture patients. Therefore, the following research questions will be addressed:

1. What is the nature and scale of adverse events and avoidable harm in the care chain for elderly hip fracture patients?

2. How is it, in the total care chain of elderly hip fracture patients, possible to reduce risks and unintended harm, and to ensure that complex care processes proceed more safely? This research question is divided into sub-questions for the three interventions:

a What is the effectiveness of the SBAR communication tool on unintended events and avoidable harm during hospital stay? (Intervention A)

b What is the effectiveness of a patient safety card with guidelines for patients and their families on signalling errors and patient trust in health care? (Intervention B)

c What is the effectiveness of evidence based bundles with regard to unintended events in the period after discharge? (Intervention C)

## Methods/Design

### Study design

This RCT is set up as a multi-centre intervention study in which hip fracture patients aged 65 years and older will be included. Surgical wards of three hospitals will participate in the intervention groups; one surgical ward in another hospital will serve as a control group. The patients in the control group receive care as usual. A baseline measurement using a retrospective record review study will be conducted. In the following RCT the effects of three different interventions will be compared to usual care, randomisation is at patient level. Within the three intervention wards patients are randomly assigned to one of four intervention groups. The groups will receive the following interventions:

• Group 1: use of SBAR communication tool for nurses in the surgical wards

• Group 2: SBAR and patient safety card

• Group 3: SBAR and information leaflet (bundles) for after discharge

• Group 4: SBAR, patient safety card and information leaflet (bundles) for after discharge

**Figure 1:**
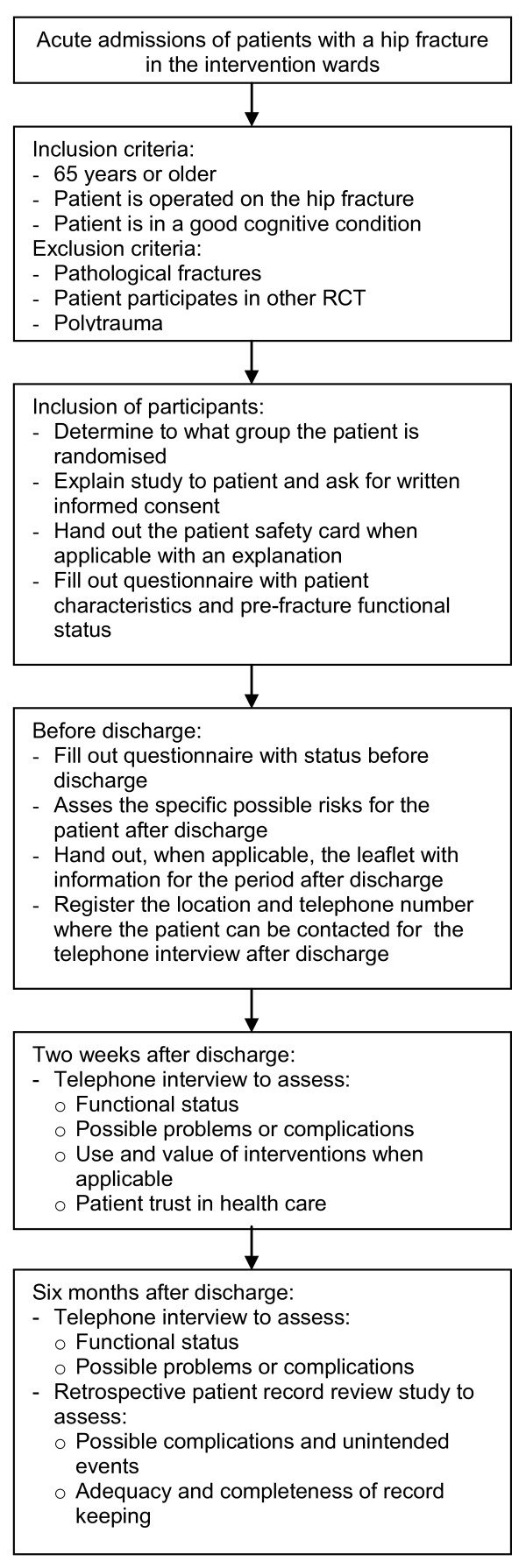
**Flow-chart of patient inclusion and data-collection**.

During the study period the morning rounds will be observed once a month in each intervention ward to look at the information transfer and communication between care providers. Two weeks and six months after discharge patients will be interviewed about their recovery and the added value of the interventions. Six months after discharge the records of the included patients will be reviewed to detect possible adverse events and unintended harm. Figure 1 gives an overview of the inclusion and data collection.

### Definitions

The definitions used in this study were adopted from previous international studies [9-11,13,33-37], Table 1 gives an overview of the used definitions.

**Table 1:** Definitions

An **adverse event **is an unintended injury that results in temporary or permanent disability, death or prolonged hospital stay and that is caused by health care management rather than by the patient's underlying disease process.
A **complication **is an unintended and unwanted event or state during or following medical specialist treatment, that has an unfavourable effect on the health of the patient to such an extend that adjustment of the medical treatment is necessary or that irreparable harm has occurred.

A **near miss **is defined as an act of commission or omission that could have harmed the patient but was prevented from completion through a planned or unplanned recovery.

**Disability **refers to temporary or permanent impairment of physical or mental function attributable to the adverse event (including prolonged or strengthened treatment, prolonged hospital stay, readmission, subsequent hospitalisation, extra outpatient department consultations or death).

**Causation **refers to injury caused by health care management including acts of omission (inactions) i.e. failure to diagnose or treat, and acts of commission (affirmative actions) i.e. incorrect diagnosis or treatment, or poor performance.

**Health Care management **includes the actions of individual hospital staff as well as the broader systems and care processes. Health care management is any care related activity that involves the delivery of care or monitoring of health which is provided by individuals or a team of professionals.

A **preventable adverse event **is an adverse event resulting from an error in management due to failure to follow accepted practice at an individual or system level. Accepted practice was taken to be 'the current level of expected performance for the average practitioner or system that manages the condition in question'.

### Informed Consent and Ethical approval

Eligible patients receive an Informed Consent form with information about the study as soon as possible after admission. Participation in the study is voluntary. Patients are only included in the study after being informed about all the aspects of the study and giving their consent to participate. The signed Informed Consent form will be returned to the researchers to allow further contact during the study. Patients are explicitly informed about the fact that they can terminate their participation in the study at any time without a specific reason and without any negative consequences for future medical treatment. Participants also have the opportunity to consult a physician not involved in the study with questions.

The privacy of the participating patients will be protected; data will be kept separated from patient names. All confidential information will be treated according to the medical confidentiality rules. Each patient will have a specific research code that is not directly traceable to a patient name; these codes are only available to the study researchers. Data related to the study will be stored on a protected server and can only be accessed by authorised members of the research team.

The study protocol is approved by the Medical Ethical Committee of the VU university medical center in Amsterdam.

### Participating surgical wards

Because the researchers will frequently have to visit the participating surgical wards during the study, one hospital region in the Netherlands will be chosen for the recruitment. Although the treatment for hip fractures is fairly standardised by means of protocols, we aim to include a surgical ward of each of the three hospital types that exist in the Netherlands; a university, a tertiary medical teaching and a general hospital. A fourth surgical ward in another hospital will be included as a control group. An inclusion criterion for the surgical wards is that they have to treat at least 100 elderly patients with a hip fracture each year. The wards will be recruited by informing already existing contacts within the wards about the study. If the ward agrees to participate an official intake by the researchers will follow. The nurses in the participating wards will be informed about the study during a meeting. In this meeting the interventions and the inclusion process will be explained. For each ward background information will be gathered about the number of patients, treatment procedures and discharge planning. This will make it possible to compare the wards and correct for important differences.

### Participants

The following inclusion criteria will be used to select eligible patients for participation in the study:

• The patient is acutely admitted to the surgical ward with a hip fracture during the research period;

• The patient is not admitted with polytrauma;

• The hip fracture is not a pathological fracture;

• The patient is 65 years of age or older;

• The patient will be operated on the hip fracture;

• The patient does not participate in an interfering study;

• The patient is in a good cognitive condition to fully understand the information about the study and to give Informed Consent.

Patients will be recruited as quickly as possible after admission to the hospital. The patients will be included by the nurses working in de wards, this process will be closely monitored by the researchers. When a patient is eligible for participation the study will be explained to them and Informed Consent will be asked. If the inclusion by the nurses does not work out as intended, the researchers will have to take over this part of the data collection.

### Randomisation

**Figure 2:**
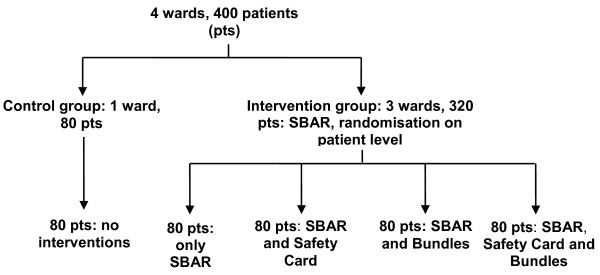
**Flow-chart of randomisation procedure**.

In this study surgical wards in three different intervention hospitals and one surgical ward in a control hospital will participate. The eligible patients in the intervention wards will be randomly assigned to one of four intervention groups. Since the SBAR tool cannot be randomised on patient level due to practical reasons, this intervention will concern all patients in the intervention wards. Before the start of the inclusion all Informed Consent Forms and intervention materials will be assigned a unique study number. The study numbers are equally divided over four intervention groups, to ensure equal group sizes as much as possible. All materials for each individual number will be gathered and put into an envelope; the stack of envelopes will be kept in the ward in consecutive order of the study numbers. Each time an eligible patient can be asked to participate, the first envelope of the stack will be used. The process of randomisation is also shown in Figure 2.

### Blinding

In this trial blinding of the patients is not possible as they either do or do not receive the material belonging to the interventions, and are therefore automatically aware of the intervention group. Since the physicians will not be directly involved in the inclusion of patients they are unaware of the intervention group a patients belongs to. For the assessment of the outcomes during a telephone interview two weeks after discharge the interviewer will be aware of the intervention group a patient belongs to because specific additional questions for each intervention group will be asked to evaluate the interventions. However, the interviewer will not be informed about the background, research questions and outcome measures of this study. For the telephone interview six months after discharge the interviewer will be unaware of the intervention group that the patient belongs to. Finally, the reviewers in the retrospective record review study will be blinded, they will assess some of the outcome measures and should therefore be unaware of the interventions that a patient might or might not have received to prevent possible biases in their assessment.

### Baseline measurement (Research question 1)

#### Aim

The aim of the baseline measurement is to determine the nature and scale of unintended events in elderly patients with a hip fracture. In addition, the reviewers will also evaluate the quality of the record keeping and the completeness of the transfer of information in the patient record. This information will be used as a starting point to evaluate the effects of the intervention study.

#### Background

Retrospective patient record review is a method that has been used to study AEs in hospitals in several countries [10,13,38]. The methods and review form used in this study will be based on the Dutch Adverse Event Study [34,39]. They used a method that was based on a protocol originally developed by the Harvard Medical Practice Study, which studied the incidence of AEs in New York State hospitals in 1984 [38].

#### Method

The incidence of complications and AEs will be measured within the care chain and especially during the hospital admission of elderly hip fracture patients. In this study we look at the care chain from the moment that the patient is brought into the emergency department until six months after discharge from the hospital. Therefore the patients' hospital record will be studied in retrospect by an experienced surgeon. For the baseline measurement patient records of hip fracture patients acutely admitted in 2007, the year previous to the start of the intervention study, will be reviewed using a structured review form. In each hospital all surgical and orthopaedic admissions of patients with a hip fracture of 65 years or older will be selected. Of these selected index admissions the medical, nursing and, if existing, the outpatient records will be collected, the X-rays made during the admission will also be available. Records of patients with a pathological fracture, polytrauma or elective admission will be excluded. The main outcome variables in this part of the study are: rate of complications, adverse events, near misses and information transfer between care providers.

The patient records will be reviewed by a team of eight experienced reviewers. The selection criteria for the reviewers are:

• At least ten years post graduate clinical (surgical) experience;

• Surgical experience with hip fractures;

• Good reputation among colleagues;

• No longer than 8 years retired at the start of the study;

• Experience or affinity with analysis of incidents, complaints and errors.

Most of the reviewers have participated in a previous record review study [39] and therefore are already familiar with this type of research, terminology and methodology. Nevertheless, they will all follow a half-day training led by the research team and one experienced reviewer. During this training, the definitions, study protocol, adjusted review form and examples of AEs will be discussed. The reviewers will also receive a review manual. They will be compensated for their review activities at an hourly rate for expenses. During the review process the reviewers can ask advice from a general internist about accepted clinical practice in the specialty of internal medicine.

The records will be reviewed by one reviewer and he determines whether complications, an adverse event or a near miss have occurred during the index admission to the hospital or the six months following the index admission using a paper based structured review form. This review form will be adapted from the Dutch Adverse Event Study [34,39] and adjusted and specified for this patient group. The review form starts with general characteristics of the admission and possible complications. Next, if the surgeon determines that an adverse event is present, the review will be continued with questions about the nature and impact, location and involved specialty, classification, preventability and causes of the adverse event. The last part of the review form will be about the clarity and completeness of information in the patient record for the transfers of the patient between the different wards and specialties within the hospital.

A random sample of 10% of the records will be independently reviewed by a second reviewer to assess the variation in the review process between reviewers.

### Intervention study

Within the intervention study three different interventions will be implemented in the surgical wards as described in the study design. The three interventions have in common that they all focus on information transfer and communication within the care chain for elderly hip fracture patients. The SBAR focuses on information transfer and communication between care professionals within the ward. The patient safety card is aimed at providing a complete picture of the current situation from patient to care provider. The information leaflet is aimed at providing useful information to the patient after discharge. In the text below each intervention will be described in more detail.

#### Intervention A: Implementing the SBAR communication tool (Research question 2a)

##### Aim

To improve communication among care providers, and for nurses to provide timely and accurate information during contacts with the physician and in the record keeping.

##### Background

Previous studies have shown that a model of structured communication can improve clinical communication [22,24]. The structured communication tool that will be used in the current study is SBAR. SBAR stands for *Situation, Background, Assessment and Recommendation *and is designed to structure and improve communication between care providers, and to provide timely and accurate information. The tool describes the most relevant points that can be discussed about a patient and how to effectively communicate them during various types of contact moments between care providers. It was developed and tested in the United States and proved to be useful there [24]. SBAR can be implemented in several ways, for instance using cards, posters near the telephones in the ward, hand-off forms and so forth to create awareness and stimulate nurses and clinicians to actively use the tool in handoff or clinical communication moments.

##### Implementation

For this study the original SBAR-tool will be translated and adapted in consultation with the participating wards to ensure that the relevant topics are present. These topics will be printed onto a pocketsize, laminated card that the nurses can keep with them during their shift. This card can be used during two different communication moments between care providers. On one side of the card there will be a description of the relevant items to discuss during the morning rounds or a telephone consult between the nurse and the physician about the condition of the patient. This side of the SBAR tool is shown in Table 2. On the other side will be a description of the relevant topics for a structured and complete record keeping. The SBAR-tool will be explained, discussed and handed over to the nurses during a meeting to announce and explain the study. The card will be accompanied by an example of a paper case how to use the SBAR-tool. Nurses will be asked to keep the card with them during their shift so that they have easy access to it and to use the card when consulting a physician or during the morning rounds. Since the SBAR-tool will be given to all nurses in the intervention wards it will apply to all hip fracture patients that are admitted during the research period.

**Table 2:** Description of the SBAR communication tool

	Identify yourself (name, function). Do you have the information below available?
**S**	**Situation**
	**Situation of the patient you are calling about:**
	• State name, department and room number of patient
	• Give a short overview of the problem and state when you visited the patient

**B**	**Background**
	**Relevant information about the background *can *include the following:**
	• Date of admission and admission diagnosis
	• Date of surgery, wound status and information about drains
	• Lab results and most recent vital signs. Is the patient using oxygen?
	• Medication overview, allergies, IV fluids
	• Other relevant clinical information
	• Mental status of the patient (delirium?)
	• Code status

**A**	**Assessment**
	**What is your assessment of the situation of the patient?**

**R**	**Recommendation**
	**What is your recommendation for this situation, examples:**
	• Come and see the patient as soon as possible
	• What actions should be taken when the situation changes?

	**Write down the comment of the physician in the patient record and changes in the condition of the patient.**

##### Evaluation

The first step is to determine to what extend and under what circumstances the SBAR tool is used by the nurses. We consider the implementation successful when 60% of the nurses uses the SBAR structure during communication moments. The use and possible effectiveness of the SBAR will be evaluated with different methods. First, short interviews will be conducted with some of the nurses of the intervention wards to discuss the possible added value, reasons for using or not using the instrument and possible improvements in the instrument or implementation for the future. Second, during the research period, observations of the morning rounds will be conducted on a regular base, approximately once a month. During these observations the process and content of communication and information transfer between nurse and physician during the morning rounds will be observed. Also, for each individual patient, it will be observed whether the different relevant topics of the SBAR-communication tool are discussed or mentioned. These aspects are, when applicable: current situation of the patient, special circumstances, possible risks, required actions, timing of the actions, reporting back, and finally, is the structure of the SBAR being used. The third evaluation moment is during the retrospective patient record review of the included patients. Here, the completeness and transfer of information in the patient record will be reviewed.

#### Intervention B: Involving patients and their families (Research question 2b)

##### Aim

To involve patients more actively in their own care process.

##### Background

The second intervention consists of a card with advice for patients about how to get more actively involved in the care process. The card was developed by the Federation of Patients and Consumer Organisations in the Netherlands (NPCF). This patient safety card provides patients with short instructions and graphical images for several topics to discuss with care providers that might be relevant for the safety of their care process. These instructions are in short:

• Give all information to the care providers about your state of health;

• Ask the care provider when something is not clear to you;

• Discuss the surgical procedure with the care provider;

• Write down an overview of your medication use;

• Ask questions when your medication looks different from expected;

• Follow the instructions given by your care provider.

##### Implementation

Within the research programme, half of the included patients in the intervention wards will receive the patient safety card. The card will be given to the patients during the hospital admission with a short instruction on how to use the card.

##### Evaluation

The implementation and effectiveness of the patient safety card will be established during an interview by telephone with the patient two weeks after discharge. We will consider the patient safety card successful when 60% of the patients has used the card and found it useful. One of the topics in the interview is therefore about the use and possible added value of the patient safety card and the need and possibility for patients and their family to get actively involved. Also, the possible consequences of the use of the patient safety card on the patients' trust in health care are discussed during these interviews. Finally, during the retrospective patient record review six months after discharge the number of complications and adverse events will be determined and compared to the baseline measurement.

#### Intervention C: Reducing avoidable harm after discharge (Research question 2c)

##### Aim

To provide the patient with information and to reduce avoidable harm after discharge, such as adverse medication events, pneumonia, urinary tract infections, wound infections, delirium, falls and pressure ulcers.

##### Background

The intervention consists of standardised information using an information leaflet with "bundles" for the patient after discharge. The concept of "bundles" was developed and tested by the Institute of Healthcare Improvement in Boston [40]. A bundle is a structured way of improving the processes of care and patient outcomes. It involves a small straightforward set of practices that have been shown to improve patient outcomes. The recommendations in the "bundles' for the period after discharge are evidence based best practices [41]. Bundles were originally developed for use by health care professionals, in our study we aim to adapt this concept for use by patients after discharge. The evidence based best practices will be reformulated to make them understandable and usable for patients and they will be printed into an information leaflet. The following topics will be described in the leaflet:

• Contact information;

• Exercise recommendations;

• Medication use;

• Problems with the operation wound;

• Thrombosis;

• Pressure wounds;

• Urinary tract infection;

• Fall prevention;

• Delirium.

##### Implementation

In the intervention wards 50% of the included patients will receive the leaflet with instructions. For each included patient a short questionnaire about the health status before discharge will be filled out to determine for which complications a patient may be at increased risk. The chapters concerning these complications are checked in the leaflet to indicate the most relevant information for each specific patient.

##### Evaluation

The use of the leaflet will be evaluated by asking the patient about the added value and the usability of the bundles during a short interview by telephone two weeks after discharge. We will consider the leaflet useful if 60% of the patients says they have used the leaflet and found it useful. The effect of the bundles will be evaluated during the telephone interview two weeks after discharge and by measuring avoidable harm (e.g. adverse medication events, urinary tract infections, wound infections, delirium, falls and pressure ulcers) within 6 months after discharge. The patient will be called again around 6 months after discharge to ask about any medical problems occurring after discharge and their recovery in the past six months. Also, the patient records of all included patients will be reviewed by experienced reviewers for additional information about possible complications and avoidable harm after discharge.

### Outcome measures

The primary outcome measures in this study are the incidence of complications and adverse events, mortality rate within six months after discharge and functional mobility six months after discharge. Secondary outcome measures are length of hospital stay, quality and completeness of information transfer and patient satisfaction with the instruments.

### General data collection and follow up

After receiving informed consent a questionnaire will be filled out with patient characteristics and the pre-fracture status of the patient. Information about the status before the fracture will be gathered using the 10-item modified Barthel Index for ADL-functioning [42], the Parker and Palmer pre-fracture mobility score [43], the Short Nutritional Assessment Questionnaire [44] and the Delirium Observation Screening Scale [45]. In addition, information will be gathered about the home situation before admission. Before discharge, the questionnaire will be completed with information about the occurrence of pressure wounds, delirium, urinary tract infections, wound infections, falls during admission and possible risk factors after discharge for these complications.

All included patients will be followed for a period of six months after discharge. During this period two interviews by telephone will be held with all included patients, two weeks and six months after discharge. During these interviews patients will be asked some multiple choice questions about their current residence, physical functioning, use of health care, the occurrence of unexpected events and some open end questions about their experiences during the whole care process. In addition to these interviews, the retrospective patient record review study will be conducted to review the patient record up to six months after discharge. The methods of this record review study will be identical to the baseline measurement described earlier. Again, the reviewers will look for complications, adverse events, the quality of record keeping and written information for the transfers of the patient between the different hospital wards. This second record review study will be used to assess the effect of the complete intervention programme on the possible reduction in complications and adverse events.

### Power calculation

We expect to reduce the absolute risk of suffering from a complication by 5% by implementing the intervention programme, with a standard deviation of 10%. With an alpha of 0.05 and a power of 80% there should be 64 participants in every intervention group and in the control group. The aim is to include 80 patients into every intervention group to compensate for possible drop-out during the follow-up phase of the study. The total number of patients included in the four different intervention groups will then be 320. In addition, 80 patients will be included into the control group.

### Data analysis

The data will be analysed using Stata 10.1 for Windows. During the data collection regular data checks (identify out-of-range answers, inconsistent responses and missing data) will be performed.

In the evaluation of the effectiveness of the intervention programme all analyses will be performed at patient and group level. The data will be analysed using the intention-to-treat principle. This means that patients will remain in the group to which they were randomly allocated at baseline. The patient characteristics will be compared for all groups and, if necessary, we will correct for differences.

The determination of complications is based on the complication registration of the Dutch Society of Medical Specialists (in Dutch: Orde van Medisch Specialisten). The determination of adverse events is based on three criteria; 1) an unintended (physical and/or mental) injury which 2) results in temporary or permanent disability, death or prolongation of hospital stay, and is 3) caused by health care management rather than the patient's disease. To determine whether the injury is caused by health care a six-point scale will be used, causation scores of 4-6 will be classified as adverse events. The preventability of the adverse events will also be measured on a six-point scale, scores of 4-6 will be classified as preventable adverse events. The incidence and mean number of complications and adverse events will be calculated.

The inter-rater reliability of the review process will be expressed as a kappa statistic. This kappa statistic will be calculated for the determination of the presence or absence of complications and adverse events. The records from the reliability study will be used for this analysis, they represent up to 10% of all reviewed patient records.

## Discussion

### Strengths and limitations of the study

One of the strengths of this study is the integrated intervention programme that will be implemented in three surgical wards. All three interventions are aimed at improving a different aspect of the care for elderly hip fracture patients. This approach may have more impact than implementing three separate interventions in different situations and could increase the possibility of finding measurable effects in patient outcomes. The randomisation at patient level reduces the influence of the differences between the three wards. In every participating ward the included patients should be equally distributed over the four intervention groups. Another important aspect of this intervention programme is that it focuses on one specific patient group that will increase in the future. The baseline measurement will give insight into the complications and adverse events that affect this patient group. This information will provide valuable insights and possibilities for improving patient safety. Finally, the follow-up of six months enables us to assess the functional recovery of patients and their experiences during those months. These experiences can be used to improve the care after discharge for this patient group and to explore the possibilities for care that is more adapted to their needs.

The study also has some limitations. First, although the results of previous studies showed that the instruments used in retrospective record review studies are sensitive for identifying adverse events [46,47], there are some important factors that have to be considered. The retrospective patient record review method relies exclusively on the information available in the hospital patient record, this can be insufficient for adverse event determination [13,48]. Also, adverse events revealed after discharge are only found if they result in a readmission or if it is recorded in the outpatient record of the patient. The hospital record is not always complete with the risk of missing important information. A general possible weakness in retrospective review studies is hindsight bias [49]. Knowing the outcome and its severity may influence judgement of causation and preventability. A second limitation of the study is that elderly hip fracture patients suffering from dementia cannot be included into the study due to practical and ethical reasons, which may lead to an underestimation of the experienced problems and therefore the possible effects of the interventions. Some other issues that might influence the outcomes in this trial are the randomisation process, the blinding of patients and the possible effect of the presence of the researcher in the intervention wards. It was mentioned before that randomisation will be on patient level. It is possible that two patients in the same room receive different interventions and expose each other to the interventions. Another possible source of bias is that patients cannot be blinded, they are automatically aware of the intervention group that they will be in as they immediately receive the interventions after inclusion. A third issue is the presence of the researchers on the wards on a regular basis for the observations. Once a month the morning rounds will be observed to assess the effectiveness of the SBAR communication tool, this in itself can increase the use of the tool.

### Feasibility

The feasibility of the study depends on a number of factors. First, we are relying on the nurses to include the patients into the trial as they are the first to know when an eligible patient is admitted to the ward. This way, the inclusion can take place as soon as possible after admission. After a few weeks it will become clear whether this method works or not. If the patients are not included by the nurses, the researchers will take over the inclusion process. Second, the success of the intervention programme will depend on the number of available patients, their decision to participate and the loss to follow-up.

### Relevance of the study

The increasing incidence of hip fractures in vulnerable elderly patients in combination with the complex care process they have to go through, with the risks of sub-optimal communication and unintended events, all contribute to the relevance of this study. This study not only aims to improve clinical communication between care providers and involve the patient more in their own care process but will also generate more knowledge and insight into the nature of adverse events and avoidable harm for this patient group. The combined use of three interventions for improving patient safety in this patient group is an innovative aspect of this study.

## Competing interests

The authors declare that they have no competing interests.

## Authors' contributions

HM wrote the manuscript, prepared the instruments for the study and was involved in the data collection. SL also prepared the instruments for the study, was involved in the data collection and critically reviewed this manuscript. IW was involved in the data collection and the critical revision of this manuscript. PJ has been involved in revising the article critically for important intellectual content. CW obtained research funding, was involved in the design of the study and critically revised the manuscript for important intellectual content. All authors read and approved the final manuscript.

## Pre-publication history

The pre-publication history for this paper can be accessed here:

http://www.biomedcentral.com/1472-6963/11/59/prepub
